# Phosphoproteome of the Oleaginous Green Alga, *Chlorella vulgaris* UTEX 395, under Nitrogen-Replete and -Deplete Conditions

**DOI:** 10.3389/fbioe.2018.00019

**Published:** 2018-03-06

**Authors:** Michael T. Guarnieri, Alida T. Gerritsen, Calvin A. Henard, Eric P. Knoshaug

**Affiliations:** ^1^National Bioenergy Center, National Renewable Energy Laboratory, Golden, CO, United States; ^2^Computational Science Center, National Renewable Energy Laboratory, Golden, CO, United States

**Keywords:** microalgae, biofuels, phosphoproteome, lipids, *Chlorella vulgaris*

The unicellular green alga, *Chlorella vulgaris* UTEX 395, represents a promising biocatalyst for renewable biofuel production due to its relatively rapid growth rate and high lipid accumulation capacity (Guarnieri et al., 2011, 2012; Gerken et al., 2013; Griffiths et al., 2014; Zuniga et al., 2016). Prior analyses have unveiled the global proteome dynamics of *C. vulgaris* following nitrogen depletion, which induces a high lipid accumulation phenotype (Guarnieri et al., 2011, 2013). More recently, we have reported a draft genome, genome-scale model, and nitrosoproteome for this alga (Zuniga et al., 2016; Henard et al., 2017)^1^ providing further insight into lipid biosynthetic-, nutrient response-, and post-transcriptional-regulatory mechanisms. To further our understanding of these regulatory mechanisms and expand the knowledge base surrounding this organism, comparative phosphoproteomic analyses were conducted under nitrogen-replete and -deplete conditions to identify differentially phosphorylated proteins that will aid in the evaluation of the potential role of phosphoregulation in lipogenesis.

## Methods

### Algal Cultivation

*Chlorella vulgaris* UTEX 395 was cultivated in biological triplicate, as described previously (Guarnieri et al., 2011, 2013). Briefly, algae were inoculated at OD_750_ = 0.05 in 1 L Roux bottles using modified Bold’s Basal Media (mBBM). Cultures were maintained at 25°C ± 1°C, with continuous (24 hr) white fluorescent light illumination (200 μE m^−2^ s^−1^). Cultures were supplemented with 2% CO_2_/air and mixed with a magnetic stir bar at 500 rpm. 50 mL of nitrogen-replete cell culture was harvested at OD_750_ = 2, centrifuged for 5 min at 5,000 × *g*, and immediately quenched on liquid nitrogen. To induce nitrogen deprivation, the remaining cell culture was centrifuged for 5 min at 5,000 × *g*, washed once in nitrogen-free mBBM, and resuspended in nitrogen-free mBBM for continued growth to OD_750_ = 4, followed by harvest, as described above. Cell pellets were immediately quenched in liquid nitrogen.

### Protein Isolation

Cell pellets were thawed and solubilized on ice in 2 mL of lysis buffer [50 mM Tris, pH 8.0, 150 mM NaCl, 1 mM DTT, 10% glycerol, supplemented with 1 × PhosSTOP Phosphatase Inhibitor Cocktail (Roche Diagnostics Corporation)]. The cells were then sonicated on ice at 4°C, at 90% power setting for 30 s × 6 cycles, with a 1 min cool-down period between sonication cycles using a Braun Sonic-L ultrasonicator. Lysates were cleared *via* two cycles of centrifugation at 16,000 × *g* at 4°C for 30 min, and the supernatants were isolated for use in subsequent phosphoproteomic analysis.

### Peptide Digestion and Phosphopeptide Enrichment

500 µg of lysate material was digested in solution with the following concentration of buffer and enzymes: 100 mM DTT, 20 mM HEPES, 100 mM iodoacetamide, 25 ng/µL trypsin (final E:S ratio of 1:20), and 20.5% TFA. 200 µg of peptide were desalted using solid phase extraction on a Waters C18 Sep Pak 1cc cartridge (part no. WAT054960) as follows: column was activated with 1 mL 100% acetonitrile followed by 1 mL 50% acetonitrile, and equilibrated with 3 × 1 mL 0.1% TFA. The sample was loaded on column and washed 2× with 1 mL 0.1% TFA, followed by elution with 0.5 mL 60% acetonitrile, 0.1% TFA. The eluate was dried to completion and phosphopeptides were enriched using the GL Sciences TiO2 kit (5010-21312), following manufacturer’s instructions. Samples were dried to completion and resuspended in 120 µL 0.1% TFA.

### Phosphoproteomic Analysis

Each enriched sample was analyzed by nano LC/MS/MS with a Waters NanoAcquity HPLC system interfaced to a ThermoFisher Q Exactive. The acquisition order was randomized. In each case, peptides were loaded on a trapping column and eluted over a 75 µm analytical column at 350 nL/min; both columns were packed with Jupiter Proteo resin (Phenomenex), employing a 2 h gradient, as follows: buffer A—0.1% formic acid in water; buffer B—0.1% formic acid in acetonitrile; t0 = 2%B, t1min = 5% B, t95 = 25% B, t110 = 35% B, t112 = 90% B, t113 = 2%B, t120 = 2%B. The mass spectrometer was operated in data-dependent mode, with MS performed in the Orbitrap at 70,000 FWHM resolutions and MS/MS performed using HCD and product ions detected in the Orbitrap at 17,500 FWHM resolutions. The 15 most abundant ions were selected for MS/MS.

### Data Processing

Data were processed through the MaxQuant software v1.5.1.0 (www.maxquant.org) for MS data recalibration, database filtering to 1% false discovery rate (FDR), calculation of peak areas (label-free), and assignment of phosphosite localization probability. Data were searched using a local copy of Andromeda with the following parameters: (i) Enzyme: trypsin/P, (ii) Database: Cv395_Maker_7100 (concatenated forward and reverse plus common contaminants), (iii) Fixed modification: carbamidomethyl (C), (iv) Variable modifications: oxidation (M), acetyl (protein N-term), phospho (STY), (v) fragment mass tolerance: 20 ppm. The Phospho(STY)Sites output was further processed using Excel. A total of 691 phosphosites (considering positional variances within the same peptide) were detected at 1% FDR.

### Data Analysis

All analysis of intensities utilized the R language (r-project.org). Proteins with no blastx identification were removed from the analysis. Intensities were filtered for presence in two of three replicates to remove erroneous measures and then compared for strict presence/absence in the two conditions. The filtered dataset of raw intensities was then analyzed for differential expression using the edgeR package (Robinson et al., 2010) and the top differentially expressed genes (*p* < 0.05) were analyzed further for gene ontology (GO). GO identifications for each protein were obtained in tab-separated format from the UniProt web interface (uniprot.org) and parsed in R to compare the top GO terms in the control replete cells versus the depleted cells. GO terms were split into molecular function, cellular component, and biological process categories when comparing between the treatments.

### Phosphoproteome Data Deposition

A master file with the full list of phosphosites and associated localization and statistical data is deposited at the FigShare repository, along with differential expression and GO analyses. All files are accessible at: https://figshare.com/s/f870b1fc4c9896f4ef81.

## Author Contributions

Project planning and wet lab execution were conducted by EK and MG. AG and CH directed phosphoproteome data processing and handling. MG and AG wrote the manuscript.

## Disclaimer

The views and opinions of the authors expressed herein do not necessarily state or reflect those of the United States Government or any agency thereof. Neither the United States Government nor any agency thereof, nor any of their employees, makes any warranty, expressed or implied, or assumes any legal liability or responsibility for the accuracy, completeness, or usefulness of any information, apparatus, product, or process disclosed, or represents that its use would not infringe privately owned rights.

## Conflict of Interest Statement

The authors declare that the research was conducted in the absence of any commercial or financial relationships that could be construed as a potential conflict of interest.

## Footnotes

^1^Guarnieri, M. T., Levering, J., Zengler, K., Henard, C. A., Boore, J. L., and Knoshaug, E. P. (under review). Genome sequence of the oleaginous green alga, *Chlorella vulgaris* UTEX 395. Submitted to Bioengineering and Biotechnology.
